# Improved Antioxidant Status after Diet Supplementation with Novel Natural-Based Supplement in Combat Athletes

**DOI:** 10.3390/sports12090247

**Published:** 2024-09-07

**Authors:** Adrian Tirla, Simona Ioana Vicas, Teodora Bianca Sirca, Corina Cinezan, Simona Cavalu

**Affiliations:** 1 Doctoral School of Biomedical Sciences, Faculty of Medicine and Pharmacy, University of Oradea, P-ta 1 Decembrie 10, 410087 Oradea, Romania; adriantirla18@gmail.com (A.T.); sircateodora@yahoo.com (T.B.S.); 2 Faculty of Environmental Protection, University of Oradea, 26 Gen. Magheru Street, 410048 Oradea, Romania; svicas@uoradea.ro; 3 Bihor County Clinical Emergency Hospital, 410167 Oradea, Romania; 4 Faculty of Medicine and Pharmacy, University of Oradea, P-ta 1 Decembrie 10, 410087 Oradea, Romania

**Keywords:** *Aronia melanocarpa*, bee pollen, sports performance, antioxidants, body composition, combat sports

## Abstract

Background: Intense physical activity is known to generate high levels of oxidative stress, and supplementation with bioactive products with powerful antioxidant effects is often recommended. In this context, the present study aims to evaluate the potential benefits of supplementing the diet of professional combat fighters with a new bioactive formulation based on *Aronia melanocarpa* (black chokeberry) and bee pollen, a natural combination with a balanced ratio of macro- and micronutrients, rich in proteins and polyphenols. Methods: A total of 31 professional combat fighters were selected to participate in this study. Due to allergic medical history, two were excluded, and the remaining 29 were divided into two groups: 14 in the control and 15 in the supplementation group. The supplemented group consumed daily 40 g of novel supplement based on a combination of dried black chokeberry and bee pollen (1:2 ratio) for 4 weeks. Results: A statistically significant increase in plasma antioxidant capacity was observed in the supplemented group compared to control related to the time and group factors at the end of this study. During this study, a significant increase in neutrophils was recorded in both groups. Supplementation with black chokeberry and bee pollen generated no significant modifications in inflammatory markers, body composition, glycemia, maximum aerobic capacity, blood glycemia, hemoglobin or red cell line. Conclusion: This clinical study pointed out a positive result in terms of plasma antioxidant capacity upon administration of the novel nutraceutical formulation.

## 1. Introduction

Elite athletes are expressly concerned about finding a diet to sustain their intense activity and often use different forms of functional foods to accomplish this goal. Multiple sports supplements are available on the market, including sports foods, medical supplements and functional foods, aiming to aid the classic diet in terms of micro- and macro-elements adapted to the necessities of each individual. In our study, we chose to utilize a combination of two natural products with multiple benefits, chokeberry and bee pollen.

Chokeberry possesses a high content of polyphenols, including procyanidins, anthocyanidins and phenolic acids, showing multiple benefits in terms of cardiovascular, neurologic and metabolic function [1]. Bee pollen, on the other side, brings a great amount of flavonoids and polyamines such as spermidine, along with carbohydrate (CHO) and protein content, essential elements in sustaining sports activity [2,3,4].

Intense physical activity is known to generate high levels of oxidative stress, and supplementation of bioactive products with powerful antioxidant effects might be sometimes recommended for a faster recovery. A novel nutraceutical formulation proposed in our previous work [2] demonstrated an intense antioxidant effect in vitro, being also a rich source of protein and carbohydrates. All these characteristics are desired in athletes’ diets and might positively impact their overall performance.

In the context of a huge market for dietary supplements and a great interest in natural ones, our work aims to highlight the effects of a natural combination and balanced ratio of macronutrients and micronutrients, rich in proteins and polyphenols. The combination of *Aronia melanocarpa* (black chokeberry), a berry with multiple health benefits, and bee pollen was demonstrated to provide multiple bioactive compounds, with a possible effect in supporting physiological adaptation while minimizing the stress generated by intense physical activity [1,5]. This clinical trial aims to evaluate in vivo the potential beneficial or unwanted effect of supplementation with a novel nutraceutical based on dried chokeberry and bee pollen.

## 2. Materials and Methods

The development of novel sports supplements based on dried chokeberries (*Aronia melanocarpa*) and multi-floral bee pollen was previously reported by our research group [2], offering personalized formulations designed to fulfill the individual requirements of different categories of athletes. According to our previous findings, a formulation with a 1:2 ratio of chokeberry/pollen demonstrated maximum synergism in terms of antioxidant capacity while providing 24.39% CHO and 17.45% proteins, elements that might help the organism to cope with intense and repeated training sessions. The raw materials were produced by professional certified farmers in Bihor County, Romania, and purchased directly from them. Hence, in the present study, we used this complex nutraceutical supplement, administered to professional athletes, in order to evaluate the clinical outcomes and health benefits of this novel formulation.

### 2.1. Subjects and Study Design

**Figure 1:**
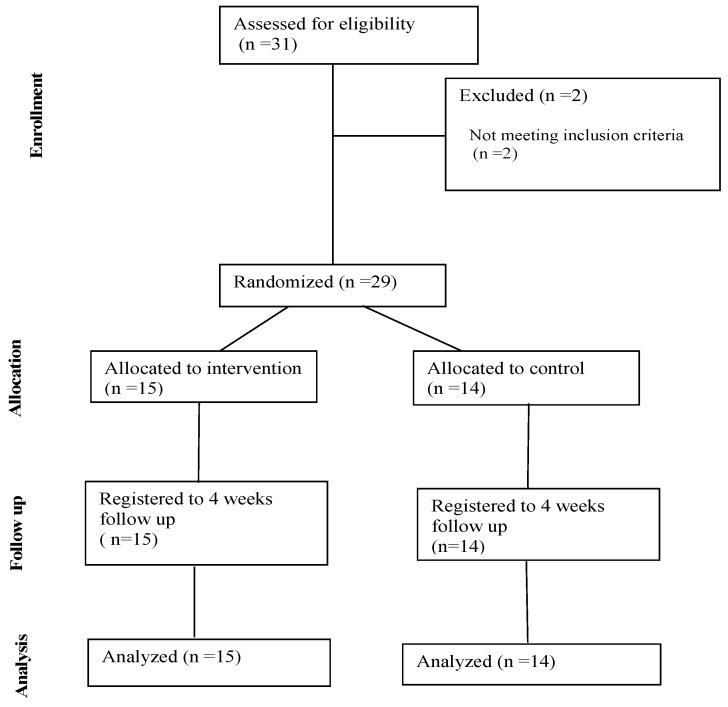
Study flowchart.

A total of 31 professional male combat fighters with an age interval of between 18–38 years were selected to take part in this study. A sports medicine doctor examined all participants before admission. Inclusion criteria referred to male Caucasian national- or international-level combat athletes with no disease history or allergies. Due to allergic medical history, two participants were excluded, and the remaining 29 were randomized using block randomization into two groups: 14 in the control group and 15 in the supplemented group (Figure 1). Each participant filled out a medical history questionnaire and had their health screened before this study started. The participants received a comprehensive explanation of the study protocol. All study procedures were carried out according to the World Medical Association (WMA) Helsinki Declaration and its amendments (Ethical Principles for Medical Research Involving Human Subjects). This study was approved by the Ethics Commission of Scientific Research within the University of Oradea, Faculty of Medicine and Pharmacy (CEFMF/03 31.10.2022). Participation in this study was voluntary, and written informed consent was obtained from all participants for accurate collection of information and data processing.

During this study, the diet of the supplemented group was augmented with 40 g of nutraceutical supplements (1:2 ratio of Aronia/pollen) per day, split into two administrations (20 g), before each training session, for 4 weeks. Considering that the flavor of our supplement was quite intense and specific, we decided that the use of placebo supplementation would not provide any benefits to our study. All participants continued their normal daily routine, which consisted of one morning physical training of 80 min in a gym supervised by a professional trainer and one evening specific training of 80 min guided by internationally accredited trainers.

All participants were evaluated in terms of VO_2_ max [6], total blood count, glycemia [7], body composition [8], inflammatory markers [9,10] and plasma antioxidant capacity [11] before and after 4 weeks. Body composition measurements (fat percentage and fat-free mass) were performed with a Body Impedance Analyzer (BIA technology), using the apparatus Tanita MC 580 connected to PC GMON Pro software version 3.4.5. VO_2_ max estimations were evaluated following the Astrand–Rhyming protocol using the Cycle Ergometer in the Sports Medicine Clinic, Bihor County Hospital, which is equipped with a Monark Ergomedic 828E Cycleergometer [6]. The heart rate recorded after the 6-min test was correlated to Astrand–Rhyming nomogram and so obtained the maximum aerobic capacity for the selected load per minute. For a fair comparison, we divided the maximum aerobic capacity by body mass to obtain the value relative to body mass.

### 2.2. Blood Collection

Blood samples were manipulated in an accredited private clinic and analyzed using Mindray BC 5300 (Mindray Nanshan, Shenzhen 518057, China) equipment for complete blood count, Ceveron M2 (Technoclone Herstellung von Diagnostika und Arzneimitteln GmbH Vienna I Austria) for fibrinogen measurements, Autolyser (DIALAB GmbH, Neudorf, Austria) for glycemia and manual methods for ESR and CRP [1,8,9]. All measurements were performed in the morning (08.00–10.00 a.m.). Blood samples were collected (10 mL) by venous puncture technique, into a vacuum tube (BD Vacutainer K2) containing 7.2 mg of EDTA. The initial sample was obtained prior to starting this study. The second sample was collected after 4 weeks. The tubes were kept in ice during transportation to the laboratory for separation. The plasma was obtained through centrifugation at 1500 rpm for 20 min at 4 °C. The collected plasma was used immediately for the biochemical and antioxidant capacity.

### 2.3. The Antioxidant Capacity—TEAC Assay

The total antioxidant capacity of blood plasma was assessed by measuring the ability of antioxidants to neutralize the blue-green-colored ABTS•^+^. The assay was carried out as described by Fischer al. [11], with some modifications. Plasma deproteination was performed by mixing plasma with an equal volume of a 10% (*w*/*v*) solution of TCA (trichloroacetic acid). After 5 min on ice, to finish deproteinating, plasma samples were centrifuged for 5 min at 14,000× *g* and 4 °C. The cation radical (ABTS•^+^) was generated by combining 7 mM solution of ABTS (2,20-azinobis-(3-ethylbenzothiazoline-6-sulfonic acid)) with 2.45 mM potassium persulfate. The mixture was then kept in the dark at room temperature for a duration of 12 h. The ABTS stock solution was diluted with phosphate buffer pH 7.4 to achieve an absorbance of 0.70 ± 0.02 at a wavelength of 734 nm. The antioxidant capacity was measured by adding 25 µL of deproteinated plasma to 2.5 mL of diluted ABTS solution and monitoring the reaction for 1 min at a wavelength of 752 nm. A calibration curve was drawn using different concentrations of Trolox standards (0.1–0.5 µM).

### 2.4. Statistical Analysis

Results were expressed as mean ± SD. Data were analyzed using 2 × 2 (group × time) mixed factorial ANOVAs to identify the presence of any main and interaction effects. The significance level threshold was set to be α < 0.05. Effect size (Cohen’s d) was determined for all outcome measures using the following formula: d = (Change_supplemented_ − Change_control_)/SD_pooled_. According to the literature [12,13], the values of effect size can be classified into small (d < 0.20), medium (0.2 < d < 0.50), large (0.5 < d < 0.80) and very large (d > 0.80). The interpretation and analysis of experimental data were performed by GraphPad Prism 9.3.0 for Windows.

## 3. Results

After 4 weeks, all 29 participants were randomized and completed the follow-up. Table 1 illustrates the age stratification of the participants within the groups.

A significant time main effect was noticed for NEU%, suggesting an increase in NEU% within groups across the intervention from the initial to the final time point. The group main effect was statistically significant for MON%, BAS% and PLT, suggesting some differences between the two groups. No statistically significant interaction Time × Group (Tx×G) was noticed for the total blood count results (Table 2).

Table 3 presents the evolution of inflammatory markers from the baseline to the end of this study. No statistically significant modifications were recorded for inflammatory markers in the supplemented or control group, except for the group main effect of ESR, which points out significant differences between the groups.

The glycemic index evolution of the participants throughout this study is presented in Table 4. Significant differences between the groups are illustrated by the main effect of the group factor.

There was no significant modification of weight, fat% or fat-free mass%, except for the BMI differences between the two groups, as presented in Table 5.

No significant time or interaction effects resulted from the VO_2 max_ determinations, but significant differences between groups were noticed, as presented in Table 6.

The results of the TEAC assay are presented in Table 7, showing a significant increase in plasma antioxidant capacity for the time and group factors, as well as for the interaction effect, showing a very large effect size.

## 4. Discussion

To our knowledge, this is the first study investigating the effects of chokeberry and bee pollen supplementation in athletes. Our study evaluated several parameters considered to be important for the well-being of the athletes, their overall health and performance. We also monitored any unwanted effects, and, fortunately, no significant adverse reactions were noticed.

In terms of NEU%, the time factor returned significant differences initial vs. final (*p* = 0.002 and medium effect size), predominantly due to a higher neutrophil/lymphocyte ratio (NLR). These results are similar to those found in elite female rowers undergoing a high-protein and low-CHO diet, published by Nieman et al. [14]. A high-protein diet resulted in increasing NLR values when compared to a high-CHO diet, in master triathletes [15]. The supplementation with novel milk-protein peptide generated a biphasic effect regarding NLR, with a significant initial decrease after the first 3 weeks, but after 6 weeks the results exceeded the initial values [16]. A study aiming to evaluate the effects of *Aronia melanocarpa* juice on young football players concluded no statistically significant differences in the WBC line [17].

In our study, some differences between the two groups regarding MON%, BAS% and PLT are observed but without any significance for time or interaction effect. Bee pollen is known to be rich in allergens, and the evolution of eosinophils was expressly part of our interest. Fortunately, no modification in eosinophils was recorded, which confirms the results of some previous studies, suggesting pollen administration as a treatment for allergic reactions [18]. Red blood cell line values can be directly linked with aerobic performance, and any allowed method of improvement is of great interest. In our study, there were no significant modifications in terms of RBC, HGB or HCT in both groups. Physical activity is known to increase RBC compared with a sedentary lifestyle, but, however, our study design did not intervene in the training programs. Other antioxidant-rich products, such as pomegranate juice, had similar results of no influence on iron metabolism after supplementation in elite rowers [19].

Body composition is an essential parameter in any sports performance but especially in sports with weight categories, where the most possible active mass is desired to be packed into a limited amount of kilograms [20]. Our study failed to demonstrate a significant lowering of body fat percentage, fat-free mass or weight. Both protein and antioxidants are known to induce optimizations of body composition. Supplementation with protein is known to increase muscle mass, and previous studies suggest that protein supplementation should be associated with CHO to support the super-compensation generated by training sessions [21,22]. Administration of a moderate amount of CHO before training sessions will not alter the overall blood sugar levels or insulin sensibility but will help to prevent muscle catabolism [22]. Other studies demonstrated that antioxidants are able to lower the fat percentage [23,24] and to prevent muscle catabolism by protecting the integrity of the muscle membrane [25]. In a double-blind study, resveratrol supplementation proved to lower body-fat mass, while improving CHO metabolism [26]. Resveratrol has been proven as an exercise mimetic and muscle synthesis promotor after supplementation in type 2 diabetes patients [26]. A lower body-fat percentage was recorded in pre-meal tomato supplementation in young women [27]. Increased lipid oxidation, detrimental to CHO, was found after a single dose of spirulina supplementation [28]. Polyphenol-rich beverages consisting of 51% Aronia succeeded in influencing lipid metabolism in healthy subjects, leading to a lower calorie intake and a better body composition [29]. Bee pollen, along with honey, has been also proven to influence lipid metabolism by reducing cholesterol levels in obese patients [30]. Berry mixtures (strawberries, bilberries, lingonberries and chokeberries) ingestion lowered postprandial glucose and insulin spikes generated by white bread [31]. Caffeine and flavonoids also increased energy expenditure and lipid oxidation in adult women [32].

Chronic inflammation is incriminated as a pathogen for multiple health problems such as cardiovascular disease, diabetes, allergies, pulmonary diseases or arthritis [33]. Sports activity is known as an inflammation modulator; even if it *per se* generates inflammation as a result of intense metabolism, it lowers the level of proinflammatory agents, leading to a lower level of chronic inflammation [34]. Our study showed no statistically significant modifications in terms of the main inflammatory markers (ESR, CRP, albumin, fibrinogen) except for the ESR differences between groups. The literature offers mixed results regarding the effects of antioxidants on inflammatory markers, with a tendency toward positive effects. Chokeberry extract succeeded in reducing inflammation markers in preadipocyte cell culture [35]. Also, it has the capacity to influence gut microbiota and, consequently, to influence inflammation levels [36,37]. One month of 3 × 100 mg Aronia extract supplementation generated no significant modifications in fibrinogen levels, but, after 2 months, fibrinogen levels increased significantly [38]. Ascorbic acid, a well-known antioxidant, proved to have no effect on CRP or fibrinogen levels in patients undergoing cardiothoracic surgery [39], while polyphenols were able to reduce fibrinogen levels in hypertensive patients [40]. Further inflammation-focused trials are necessary to evaluate the possible effects of our proposed supplement on inflammation levels.

Antioxidants are often recommended to improve risk factors in diabetic patients, including the glycemic profile [41]. Our study reported differences in glycemia with respect to the group factor but no significant effect for the time and interaction. It should be taken into consideration that our study started from a normal baseline, not from a pathological one requiring correction. Other studies reported mixed results; for example, the ingestion of 200 mL of chokeberry sugar-free juice successfully lowered fasting glucose levels in non-insulin-dependent diabetic patients, during a 3-month study [42]. A more recent study evaluated the effects of 35 g of fermented and non-fermented chokeberry in type 2 diabetes mellitus patients, and no statistically significant influence was recorded in terms of waist circumference, insulin sensitivity or fasting levels of glucose, insulin or glucagon [43]. Although polyphenols have a blood sugar-lowering potential [44], and the glycemic values of participants in the supplemented group were lower, no significant time effect or interaction were recorded.

Although some previous studies pointed out that polyphenols are able to enhance aerobic capacity [45], our study showed statistically significant VO_2_ max differences between groups but no significant differences regarding time or interaction factors, despite the increased fat-free mass in the supplemented group. Other studies presented similar findings; for example, no improvement in aerobic capacity was noticed after Aronia juice supplementation in young football players [17]. Long-term (6 weeks) antioxidant supplementation resulted in lower VO_2_ max values and might blunt adaptations generated by physical activity [46]. No differences in terms of VO_2_ max were noticed after supplementing 2 g of curcumin for 6 weeks in amateur long-distance runners [47].

Intense physical activity is known to generate high amounts of ROS as a consequence of the major ergogenic processes. Exercise-induced oxidative stress can alter the pro-antioxidant balance, leading to a disbalance in the pro-oxidant/antioxidant state, especially in elite athletes, where prolonged intense activity is often encountered [48]. Our supplement based on the combination of chokeberry and pollen proved to have an intense antioxidant effect not only in vitro but also in vivo, as the plasma antioxidant capacity was statistically increased with a significant p value for both factors and a large Cohen size effect. Aronia juice alone succeeded in reducing oxidative stress in young football players [17], but, to our knowledge, there are no studies yet demonstrating the effect of bee pollen on oxidative status. Other natural products were investigated regarding plasma antioxidant capacity, such as the supplementation of pomegranate juice in elite rowers [19]. Coenzyme Q10 prevented the increase in prooxidant markers generated after supramaximal exercise in sedentary men and, after game, in soccer players [49,50].

Limitations of our study might be related to the small number of participants included and the narrow inclusion criteria, limited to male combat sports players. Other limitations might arise from the study design, consisting of limited biological markers selected to be analyzed, alongside the limitations derived from the accuracy of some methods (e.g., Astrand protocol) and the reduced number of determinations. Possible interferences with other foodstuffs or supplements might generate limitations, as we were not able the control the entire diet and physical activity of the participants. The lack of placebo supplementation in the control group generates another limitation of this study. The strength of our study consists of the novelty of the proposed supplement, based on the combination of chokeberry and bee pollen, its complexity and multi-valences, but also the relative homogeneity of the participants.

## 5. Conclusions

The novel nutraceutical formulation based on bee pollen and dried chokeberries succeeded in providing a positive result in terms of plasma antioxidant capacity in professional combat fighters. An increase in NEU% was noticed during this study in both groups. Statistically significant differences between the groups were remarked in terms of MON%, BAS%, PLT, ESR, blood glycemia, VO_2_ max, but with no significant time or interaction effect. No significant modification of aerobic capacity or body composition was recorded during our study. Overall, the novel formulation proved to be a good source of antioxidants, proteins and CHO, which are important elements desired in any athlete’s diet.

## Figures and Tables

**Table 1:** The age and practiced sports distribution (supplemented vs. control).

Characteristics	Supplemented Group	Control Group
Age (mean ± SD)	25 ± 6.34	26 ± 5.42
Practiced sport and number of participants	Judo (11) MMA* (3) Wrestling (1)	Judo (10) MMA* (2) Wrestling (1) Boxing (1)
Training program	11 training sessions per week (six specific, five physical training, each 80 min long)	

* MMA-Mixed Martial Arts.

**Table 2:** Complete blood count (WBC, NEU%, LYM%, NLR, MON%, EOS%, BAS%, RBC, HCT-%, HGB-g/dl, PLT) recorded for supplemented and control group.

Parameter	Supplemented Group		Control Group		*p*-Value Time Factor	*p*-Value Group Factor	Interaction T × G	Cohen’s *d*	Normal Range
	Initial	Final	Initial	Final					
WBC	5.50 ± 0.90	6.33 ± 0.70	6.09 ± 0.89	6.07 ± 1.12	0.0964	0.4935	0.0814	0.27	4.00–10.50 /10^3^/uL
NEU%	50.22 ± 4.5	55.90 ± 3.77	49.20 ± 7.58	54.14 ± 8.17	**0.0020**	0.3997	0.8221	0.27	45.0–76.0%
LYM%	38.53 ± 3.61	34.71 ± 3.89	36.43 ± 7.40	33.64 ± 6.65	0.2236	0.9540	0.1750	0.19	25.0–55.0%
NLR	1.51 ± 0.92	1.72 ± 0.65	1.32 ± 0.27	1.64 ± 0.28	0.1003	0.3814	0.7374	0.15	
MON%	6.96 ± 0.81	5.82 ± 0.86	8.86 ± 3.85	7.92 ± 2.49	0.0950	**0.0019**	0.8708	1.12	0.0–15.0%
EOS%	3.82 ± 1.52	3.19 ± 2.58	3.05 ± 1.56	3.38 ± 2.16	0.7773	0.5849	0.3671	0.07	0–7%
BAS%	0.45 ± 0.12	0.37 ± 0.79	1.22 ± 0.55	0.98 ± 0.35	0.2445	**<0.0001**	0.5588	0.99	0–2%
RBC	4.91 ± 0.41	5.01 ± 0.31	4.82 ± 0.41	4.89 ± 0.40	0.4033	0.3027	0.8824	0.33	4.5–5.7/ 10^6^/uL
HGB	14.59 ± 0.66	14.76 ± 0.66	14.49 ± 1.14	14.65 ± 1.04	0.4987	0.6415	0.9662	0.12	13.5–17.2/ g/dL
HCT	42.94 ± 2.08	43.71 ± 2.17	43.08 ± 3.35	44.59 ± 3.34	0.1245	0.4881	0.6146	0.31	40.0–50.0%
PLT	266.6 ± 22.85	271.5 ± 31.93	229.70 ± 50.78	248.90 ± 61.98	0.3031	**0.0131**	0.5399	0.45	150–450/ 10^3^/uL

Legend: WBC-white blood cells, NEU%-neuthrophils, LYM%-lymphocites, NLR-neutrophil/lymphocyte ratio, MON%-monocites, EOS%-eosinophils, BAS%-basophils, RBC-red blood cells, HCT%-hematocrit, HGB-g/dL-hemoglobin, PLT-platelets. Significant *p*-value is marked in bold letters.

**Table 3:** The evolution of inflammatory markers following supplementation.

Parameter	Supplemented Group		Control Group		*p*-Value Time Factor	*p*-Value Group Factor	Interaction T × G	Cohen’s *d*	Normal Range
	initial	final	initial	final					
ESR	2.86 ± 1.59	3.00 ± 1.41	6.78 ± 3.92	7.71 ± 4.92	0.5362	**0.0001**	0.6476	1.30	0–12/mm/1 h
CRP	2.26 ± 0.96	2.00 ± 0.75	2.00 ± 0.87	2.42 ± 0.64	0.7105	0.7105	0.1186	0.60	0–6/mg/L
Fibr	295.8 ± 53.57	295.5 ± 58.95	283.10 ± 46.01	300.00 ± 36.44	0.5281	0.7550	0.5134	0.09	180–450/mg/dL

Legend: ESR- erythrocyte sedimentation rate, mm/1 h; CRP-C reactive protein, mg/L; Fibr-fibrinogen-mg/dL. Significant *p*-value is marked in bold letters.

**Table 4:** Blood glycemia (mg/dL).

Parameter	Supplemented Group		Control Group		*p*-Value Time Factor	p-Value Group Factor	Interaction T × G	Cohen’s *d*	Normal Range
	Initial	Final	Initial	Final					
Blood glycemia	85.74 ± 3.58	81.93 ± 7.77	87.57 ± 8.76	89.08 ± 8.57	0.5580	**0.0253**	0.1784	0.87	70–115

Significant *p*-value is marked in bold letters.

**Table 5:** Body composition in terms of weight (kg), fat%, fat-free mass%, BMI (kg/m^2^).

Parameter	Supplemented Group		Control Group		*p*-Value Time Factor	*p*-Value Group Factor	Interaction T × G	Cohen’s *d*
	Initial	Final	Initial	Final				
Weight	87.89 ± 12.83	88.17 ± 12.50	88.54 ± 16.68	88.54 ± 16.39	0.9711	0.8951	0.9711	0.02
Fat%	15.31 ± 3.31	15.00 ± 3.15	15.79 ± 3.83	15.70 ± 3.63	0.8277	0.5216	0.9047	0.20
FFM%	84.69 ± 3.31	85.00 ± 3.15	84.21 ± 3.83	84.30 ± 3.63	0.8277	0.5216	0.9047	0.20
BMI	26.98 ± 2.91	27.07 ± 2.82	27.57 ± 3.58	27.56 ± 3.50	0.9623	0.5245	0.9529	0.15

Legend: Weight (kg); fat%; FFM-fat-free mass%; BMI -Body mass index, (kg/m^2^).

**Table 6:** Maximum aerobic capacity (VO_2 max_, ml/kg).

Parameter	Supplemented Group		Control Group		*p*-Value Time Factor	*p*-Value Group Factor	Interaction T × G	Cohen’s *d*
	Initial	Final	Initial	Final				
VO_2 max_	46.35 ± 2.82	46.66 ± 2.58	44.51 ± 3.12	44.92 ± 3.12	0.6398	**0.0230**	0.9481	0.60

Significant *p*-value is marked in bold letters.

**Table 7:** Plasma antioxidant capacity using TEAC assay.

Parameter	Supplemented Group		Control Group		*p*-Value Time Factor	*p*-Value Group Factor	Interaction T × G	Cohen’s *d*
	Initial	Final	Initial	Final				
TEAC	**0.30 ± 0.04**	**0.42 ± 0.04**	0.29 ± 0.039	0.30 ± 0.04	<0.0001	<0.0001	<0.0001	2.96

Plasma antioxidant capacity expressed as Trolox Equivalents (TE). Significant *p*-value is marked in bold letters.

## Data Availability

The data that support the findings of this study are available from the corresponding author upon reasonable request and in compliance with the General Data Protection Regulation. There are ethical restrictions on sharing data publicly, as our data sets contain potentially sensitive health-care-related information. Due to the amount of data on each patient, only pseudo-anonymization would be possible.

## Abbreviations

BAS	basophils
BMI	body mass index
CHO	carbohydrates
CRP	c-reactive protein
EOS	eosinophils
ESR	Erythrocyte sedimentation rate
HCT	hematocrit
HGB	hemoglobin
LYM	lymphocytes
MON	monocytes
NEU	neutrophils
NLR	neutrophyls/lymphocytes ratio
PLT	platelets
RBC	red blood cells
ROS	Reactive oxygen species
TEAC	trolox equivalent antioxidant capacity
VO_2_ max	maximum aerobic capacity
WBC	white blood cell
